# Metformin and blood cancers

**DOI:** 10.6061/clinics/2018/e412s

**Published:** 2018-08-28

**Authors:** Ademar Dantas Cunha Júnior, Fernando Vieira Pericole, Jose Barreto Campello Carvalheira

**Affiliations:** Departamento de Medicina Interna, Universidade Estadual de Campinas, Sao Paulo, SP, BR

**Keywords:** Metformin, Diabetes, Blood Cancers, myeloma, leukemia, lymphoma

## Abstract

Type 2 diabetes mellitus and cancer are correlated with changes in insulin signaling, a pathway that is frequently upregulated in neoplastic tissue but impaired in tissues that are classically targeted by insulin in type 2 diabetes mellitus. Many antidiabetes treatments, particularly metformin, enhance insulin signaling, but this pathway can be inhibited by specific cancer treatments. The modulation of cancer growth by metformin and of insulin sensitivity by anticancer drugs is so common that this phenomenon is being studied in hundreds of clinical trials on cancer.

Many meta-analyses have consistently shown a moderate but direct effect of body mass index on the incidence of multiple myeloma and lymphoma and the elevated risk of leukemia in adults. Moreover, new epidemiological and preclinical studies indicate metformin as a therapeutic agent in patients with leukemia, lymphomas, and multiple myeloma. In this article, we review current findings on the anticancer activities of metformin and the underlying mechanisms from preclinical and ongoing studies in hematologic malignancies.

## INTRODUCTION

Based on the increasing incidence of cancer along with increasing rates of obesity and type 2 diabetes mellitus (DM2), much effort has been made to identify the epidemiological and biological connections between these conditions. In the clinic, oncologists are gradually being required to tailor cancer treatments for patients with pre-existing diabetes or obesity, and endocrinologists often need to control diabetes in patients who are undergoing treatments for cancer 1.

Despite contradictory reports in the early literature 2-4, many meta-analyses have consistently shown a moderate but direct effect of body mass index (BMI) on the incidence of lymphoma and multiple myeloma (MM) and the elevated risk of leukemia in adults 5-8. Similarly, patients with DM2 frequently develop hematological malignancies 9-11. Notably, the incidence of tumors in patients with DM2 is profoundly affected by the treatment—long-term metformin use has been found to be connected with a reduced incidence of cancer and cancer-related mortality 2.

In this review, the association between hematological cancers and diabetes and the epidemiological, clinical, and preclinical data that establish the value of metformin as a potential treatment for hematologic malignancies are summarized.

### Diabetes and cancer

Although the link between DM2 and carcinogenesis was first reported in 1910 by Maynard and Pearson (1910 apud Wojciechowska, 2016) 10, it was over 100 years later that the *American Diabetes Association* (ADA) and *American Society of Clinical Oncology* (ASCO) presented their consensus on the factors that link diabetes and cancer 10,12. Notably, DM2 and cancer share many risk factors, especially obesity 12, and DM2 and malignancies are correlated through several conditions, such as hyperinsulinemia (due to resistance to endogenous or exogenous insulin), hyperglycemia, and chronic inflammation due to overweight and body fat mass 10,12.

Adiposity has been correlated with an increased risk of several cancers, including breast, endometrial, pancreatic, and colon cancer. In addition, obese men and women with esophageal, gallbladder, colorectal, pancreatic, liver, and kidney cancer have an elevated risk of cancer-related deaths (Table 1) 13. Notably, BMI consistently has a moderate but direct effect on the rates and risk of death due to lymphoma and MM and is associate with an increased risk of leukemia in adults (Table 2) and of monoclonal gammopathy of uncertain significance (MGUS) transforming into MM 3,6-8,3-26.

Mechanistically, obesity—a state of excess nutrient levels—chronically activates signaling pathways that upregulate insulin, insulin-like growth factor (IGF), leptin, and inflammatory cytokines (e.g., IL-6) and increases the risk of neoplastic transformation. Consistent with this model, elevated levels of adiponectin, a marker of reduction in adipose tissue, were recently found to be associated with a lower risk of MM 27,28. These factors stimulate cell surface receptors and signaling through Janus kinase (JAK)/signal transducers and activators of transcription (STATs), mitogen-activated protein kinase (MAPK), and phosphatidylinositol 3-kinase (PI3K), and all of these molecules are commonly dysregulated in neoplastic cells 29.

Conversely, studies worldwide have shown that DM2 is associated with increased rates of many cancers and cancer-related mortality (Table 3) 36-47. Notably, DM2 is linked with the incidence of solid tumors and an increased risk of lymphoma, leukemia, and MM. A meta-analysis of 26 studies observed an increased risk of non-Hodgkin lymphoma (NHL) and peripheral T cell lymphoma (PTCL), unlike other subtypes of NHL, among patients with DM2. DM2 patients have a risk of 1.22 of developing leukemia (95% confidence interval (CI), 1.03-1.44; P=0.02) and a nonsignificant high risk for developing myeloma (1.22, 95% CI, 0.98-1.53; P=0.08) 48. Further, peak postload glucose levels were correlated with an increased risk of mortality from MM, NHL, and leukemia in the study by Chiu et al. 49. A recent review concluded that the association between DM2 and various cancers is supported by strong evidence in only 14% (breast cancer, intrahepatic cholangiocarcinoma, colorectal cancer (CRC), and endometrial cancer) of the 27 correlations investigated, with no bias 50. 

As the incidence of DM2 is increasing worldwide, MM, leukemia, and NHL are being diagnosed more frequently with concomitant diabetes mellitus 9,48,51-53. Therefore, physicians who treat patients with these comorbidities must consider the possible effects of the treatments for MM and leukemia on glucose metabolism 54,55.

#### Antihyperglycemic agents and the risk of cancer

Early studies have observed that compared to no therapy, metformin monotherapy is correlated with a reduced risk of developing cancer, whereas sulfonylurea therapy is correlated with an increased risk 59. These observations led to the hypothesis that hypoglycemic agents differentially influence the risk of cancer, depending on the concentrations of insulin that they induce 60.

Although an initial meta-analysis failed to detect an elevated cancer risk in association with the use of insulin, the meta-analysis had small sample sizes and short durations of study 61,62. Consistent with the above hypothesis, a subsequent meta-analysis in larger populations demonstrated an increase in the overall cancer risk (relative risk [RR] 1.39; 95% CI: 1.14-1.70) correlated with insulin therapy 63-65.

In contrast, a recent meta-analysis reported mixed results concerning insulin secretagogues and the risk of cancer, demonstrating a correlation between the administration of sulfonylureas and an increased risk of some cancers but not others 66,67. In addition, a recent randomized controlled study of over 12,000 patients (average follow-up over six years) failed to demonstrate an increase in the rates of cancer or cancer-specific mortality in insulin users *versus* controls 68. However, a large meta-analysis of 182 randomized controlled trials including 135,540 patients with diabetes linked the use of metformin and thiazolidinediones (TZDs) with a reduced risk of cancer 69. These reviews suggest that the insulin level is not a surrogate marker of cancer development, thereby indicating a unique mechanism of metformin activity in cancer.

### Metformin, cancer, and onco-hematological diseases

#### History of metformin

**Figure 1:**
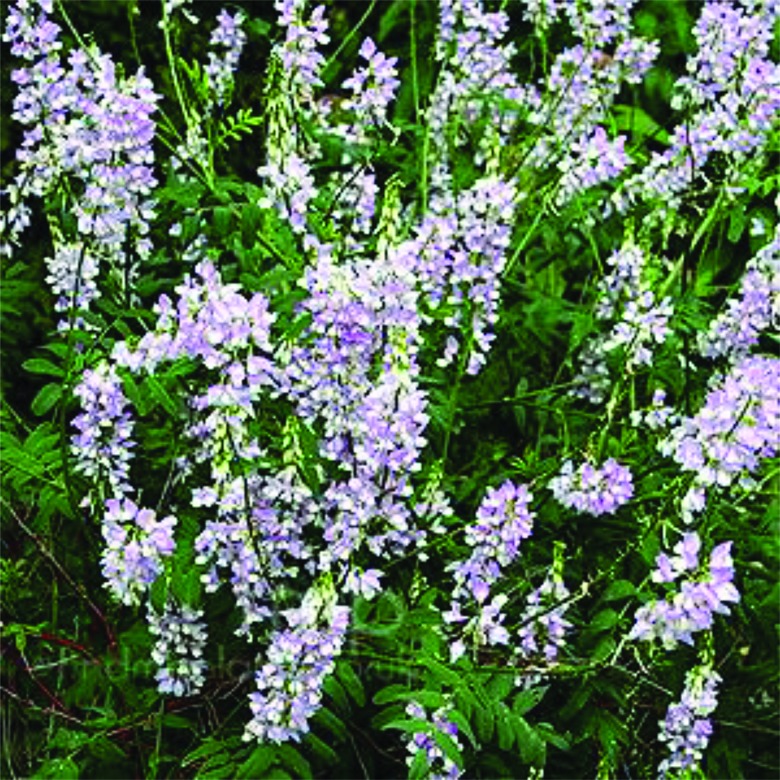
*Galega officinalis* (goat's rue). http://www.findmeplants.co.uk/photos/galega_officinalis.jpg

Metformin is a derivative of biguanide that has been used for nearly one century to treat DM2. Biguanides initially derived from *Galega officinalis* (Leguminosae) (or *goat's rue*), *French lilac*, *Spanish sainfoin*, and *false* i*ndigo* have been recognized for their medicinal value since medieval times in Europe (Figure 1) 70. *G. officinalis*—arruda-caprária in Brazil—is a medicinal plant that originated in Europe and Asia 62-64, and its medicinal properties have been recognized since medieval times, during which *G. officinalis* was prescribed in folk medicine to relieve polyuria accompanying DM2 71.

Although *G. officinalis* reduces glucose concentration in blood of patients with diabetes, the search for its active compound has been slow, primarily due to the effects of light on this plant and the introduction of insulin. Studies at the end of the 19th century indicated that *G. officinalis* was rich in guanidine, and in 1918, guanidine was shown to have hypoglycemic activity in animals. Nonetheless, guanidine was incredibly toxic for clinical use, prompting interest in galegine (isoamylene guanidine) 71.

In 1929, several biguanides that reduced serum glucose levels, including dimethyl biguanide (1,1-dimethyl biguanide hydrochloride or metformin), were synthesized. Jean Sterne (1909-1997) was the first to perform studies with galegine. Sterne selected dimethyl biguanide (metformin) for clinical development and proposed the name Glucophage. In contrast to sulfonylureas, metformin fails to induce insulin secretion but impedes the release of glucose by the liver and increases muscle glucose uptake 71.

Interestingly, the benefits of metformin in the treatment of diabetes have gained prominence only since 1995. The United Kingdom Prospective Diabetes Study (UKPDS) was a milestone project showing that regardless of glycemic control, metformin reduced the risk of myocardial infarction and all-cause mortality. Consequently, diabetes experts around the world indicated metformin as the first-choice drug for DM2 70, and metformin has become the most frequently administered agent to treat DM2 72,73. 

In 1995, the benefits of metformin in people with diabetes were found to not only be limited to glycemic control, and metformin was shown to reduce the risk of malignancy in patients with diabetes. Evans et al. 2 were the first group to note an inverse correlation between cancer and metformin use, wherein patients who were exposed to metformin had a low risk of developing cancer. Since then, various studies in DM2 patients have shown that metformin use is associated with a reduced risk of several cancers 2,11,74-79, including prostate cancer 80,81, lung cancer 45,82,83, head and neck cancer (HNC) 84,85, breast cancer 86-88, pancreatic cancer 89, CRC 90-92, endometrial cancer 93, ovarian cancer 94-96 and hepatocellular carcinoma (HCC) 97,98 (Table 4).

#### Mechanisms of action of metformin and preclinical and ongoing studies in hematologic malignancies

**Figure 2:**
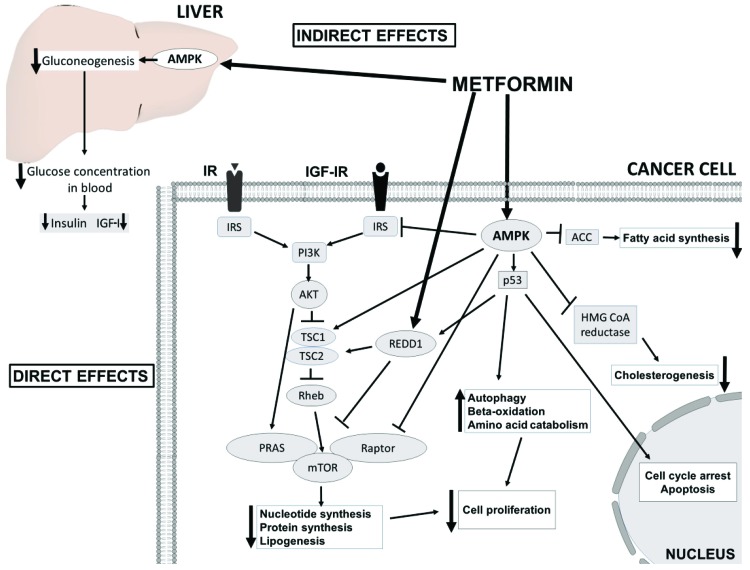
The insulin-dependent (indirect effects) and AMPK-dependent molecular mechanisms (direct effects) underlying the anticancer effects of metformin. AMPK activation in the liver results in decreased insulin and IGF-1 levels and consequent attenuated downstream signaling. In cancer cells, AMPK inhibits PI3K/AKT/mTORC1 signaling directly through the phosphorylation of the Raptor subunit and indirectly through the phosphorylation of the TSC1/2 complex and insulin receptor substrate 1 (IRS1) and the activation of regulated in development and DNA damage response 1 (REDD1). In addition, metformin-induced activation of AMPK leads to the phosphorylation of p53, inducing cycle arrest, apoptosis and autophagy. Inhibition of mTORC1 results in a decrease in global protein synthesis and lipogenesis. Metabolic alterations are also achieved by the inhibition of acetyl-CoA carboxylase (ACC) and 3-hydroxy-3-methylglutaryl-CoA (HMG-CoA) reductase.

Two potential accepted antineoplastic mechanisms of metformin have been proposed (Figure 2). First, metformin inhibits mitochondrial complex 1, resulting in low ATP production and an increase in the concentration of ADP; ADP is transformed to AMP by the catalytic effect of the enzyme adenylate kinase. Under low ATP concentrations, AMP binds to the γ subunit of�AMPK leading to some conformational changes in the α subunit, and this favors the phosphorylation of AMPK 72. Moreover, the inhibition of complex 1 produces reactive nitrogen species. These radicals stimulate protein kinase C ζ (PKCζ), which successively phosphorylates LKB1L and LKB1S, thereby leading to the activation of LKB1. Activated LKB1 phosphorylates and activates AMPK 90, resulting in the inhibition of downstream AKT/mTOR signaling and consequent suppression of cell proliferation 100,101. Second, metformin induces reductions in circulating insulin concentrations and IGF, preventing the activation of the insulin and IGF receptor signaling pathways and resulting in decreased growth promotion and mutagenesis 102,103. Notably, IGF-1 and insulin receptors interact to implement antiapoptotic signaling that increases AKT/mTOR kinase activity 104,105.

Despite the importance of LKB1 in the AMPK-dependent antineoplastic effects of metformin, this drug abolishes the increase in tumor growth linked with high-fat diet and hyperinsulinemia, irrespective of LKB1 expression by the tumor. This antineoplastic activity is connected with decreased circulating insulin levels and insulin receptor activation in tumoral tissues, similar to the mechanisms underlying tumor growth repression by dietary control 106-109.

Many studies have shown that metformin synergizes with chemotherapeutic drugs at low doses, minimizing the side effects of high doses 99,110,111. More than 40 phase I/II clinical cancer trials (http://clinicaltrials.gov/) on metformin in combination with chemotherapeutics are underway worldwide. These studies are examining the antineoplastic effects of metformin. This drug is used jointly with chemotherapeutic agents in cancers of the digestive (hepatic, gastric, pancreatic, and colorectal) and reproductive systems (ovarian and endometrial) and lung, prostate, and breast cancer, and the clinical limitations of metformin in cancer treatment are being determined. Several trials have reported synergistic or additive effects of such combinations 87,111-131. In contrast, observational retrospective studies have observed that these combinations are antagonistic in certain cancers 132-136. Therefore, metformin synergizes with standard chemotherapy drugs to increase chemosensitivity in specific cancers.

Cell metabolism is epigenetically regulated 137,138. Cells control their metabolism in response to extracellular signals and the availability of nutrients by altering their epigenetic and transcriptional programs. Notably, metformin is also involved in this cooperation between epigenetic and metabolic mechanisms. Du et al. 139 found that metformin decreases histone H2B (H2BK120) and monoubiquitination levels and inhibits the transcription of target genes, such as cyclin D1 and p21. Metformin-activated AMPK increases the histone deacetylase activity of SIRT1 to downregulate acetylated p53 and p21 levels 140. Further, the loss of the MM SET domain (MMSET) in MM cells 141 is regulated by metformin in prostate cancer cells 142. Finally, metformin decreases the levels of a histone demethylase coactivator 143 and a metabolic enzyme in the TCA cycle 144,145.

#### Myeloma and metformin

AKT mediates the pathogenesis and progression of MM and resistance to standard treatments. Many upstream signaling pathways converge on AKT to govern survival and proliferative signals and apoptosis suppression. Early data have revealed high expression of AKT in myeloma cell lines and bone marrow aspirates from patients, especially in the advanced stages 105. In accordance, *in vitro* and *in vivo*, the combination of metformin and dexamethasone synergizes to eliminate MM cells, inhibiting cell proliferation through reduced AKT/mTOR signaling 146. This phenomenon is mediated by the phosphorylation of TSC2 147, which negatively regulates cell growth by acting upstream of mTOR 148.

Notably, metformin inhibits glucose regulatory protein 78 (GRP78), an essential factor in bortezomib-induced autophagy and pharmacologically increases the anti-myeloma effects of bortezomib. Concomitant treatment with metformin and bortezomib inhibits the effects of the unfolded protein response (UPR) on GRO78, thus impairing autophagosome formation and increasing apoptosis and confirming the impeded growth of xenotransplanted myeloma cells *in vivo* 149.

The low toxicity and potent *in vitro* and *in vivo* effects of metformin, in combination with the findings of retrospective epidemiological studies in various cancer models, have prompted nearly 200 trials (ClinicalTrials.gov) examining metformin alone or in combination with other antitumoral agents in patients without diabetes. However, few studies are determining the effects of metformin in MM (Table 5).

#### Leukemias and metformin

The LKB1/AMPK/mTOR axis functions in hematopoietic cancers, such as acute myeloid leukemia (AML) and acute lymphoblastic leukemia (ALL); consequently, metformin represents a new perspective in hematological cancer therapy. Nevertheless, metformin does not appear to alter the growth, differentiation, or survival of normal CD34+ stem cells 150-152. 

Mutations/deletions in PTEN or post-translational inhibition of its lipid phosphatase activity is responsible for much of the chronic stimulation of PI3K/AKT/mTOR signaling in T-ALL 153. In accordance, deletion of LKB1 in mice on a PTEN+/- background increases the incidence of various tumors, including lymphomas, the development of which is significantly delayed by metformin 154. Thus, metformin might increase the chemosensitivity of ALL through its inhibitory effects on the AKT/mTOR pathway via AMPK activation 155,156.

Insulin might contribute to the chemoresistance of ALL cells by activating PI3K/AKT/mTOR signaling. IGF-1 signaling increases the proliferation of cell lines 157,158 and inhibits dexamethasone-induced apoptosis 158, promoting the growth of malignant cells. Thus, elevated levels of exogenous insulin might activate insulin and IGF-1 receptor (IGF-1R) on ALL blasts 157, a phenomenon that can be blocked through a metformin-mediated reduction in insulin levels.

The PI3K/AKT/mTOR pathway is also stimulated by upstream oncogenes, such as the protein kinase BCR-ABL, a t^9^,^22^ translocation product in chronic and acute Ph1+ leukemias, and the Tax oncoprotein, which is central in the pathogenesis of HTLV human retrovirus infection and adult T cell leukemia. Through the activation of AMPK, metformin suppresses the proliferation and clonogenic activity of several chronic myeloid leukemia (CML) lines, such as those that are imatinib-resistant and T315I BCR-ABL mutants 152.

In leukemic stem cells and stem cells of solid tumors, metformin is selective toward inducing death *in vitro* and tumor xenografts 159. These findings are notable because the leukemic stem cells that persist despite antineoplastic treatment are one of the causes of neoplasia relapse 160.

When combined with chemotherapy or other drugs, metformin might have additive effects on reducing cell growth and drug efflux through its activity on AMPK or P-glycoprotein 161,162. In acute promyelocytic leukemia (APL), metformin synergizes with trans-retinoic acid, inducing the differentiation and apoptosis in leukemic blasts 163. An examination of glucose dependence in chronic lymphocytic leukemia (CLL) cells demonstrated differential sensitivity to glucose deprivation; thus, the administration of the HIV protease inhibitor ritonavir and metformin to CLL cells has provided a strong rationale to target glucose metabolism and the ensuing metabolic plasticity in CLL 164.

Based on these findings, future studies should examine the role of metformin in improving chemotherapy outcomes in ALL patients with or without diabetes. Many trials are testing metformin in patients without diabetes who have solid tumors. Four recent clinical trials (ClinicalTrials.gov) are measuring the clinical and biological outcomes of metformin combined with standard systemic chemotherapy in relapsed ALL (Table 6), and their findings will be reported soon.

#### Lymphoma and metformin

Shi et al. 165 have provided the first evidence of the *in vitro* and *in vivo* activity of metformin in human lymphoma cells, demonstrating that the activity of drug on AMPK inhibits the growth of B and T cell lymphomas by inhibiting mTOR signaling without the involvement of AKT. Moreover, the response of lymphoma to drugs such as doxorubicin and temsirolimus, a mTOR inhibitor, is significantly improves upon coadministration with metformin. In addition to inhibiting the mTOR pathway, metformin activates p53 by suppressing murine double minute X (MDMX), thereby causing apoptosis 166.

Rosilio et al. 167 demonstrated that metformin, phenformin, and AICAR, an AMPK activator, have robust antitumor activities against human T-LEN and human T-ALL lines. The mechanism of action of these agents is to suppress tumor metabolism and proliferation by inducing apoptosis, activating AMPK, and constitutively inhibiting mTOR. Further, several signal transduction pathways [mTOR, AKT, NF-kappaB (NF-κB), Fatty acid synthase (FASN), and insulin-like growth factor-1 receptor (IGF-1R)] are overexpressed in Peripheral T-cell lymphomas (PTCL), supporting the use of metformin as an inhibitor of mTORC2 and NF-κB in PTCL 168.

In diffuse large B cell lymphoma (DLBCL) patients with diabetes, metformin, as a first-line chemoimmunotherapeutic agent with rituximab, improved progression-free survival (PFS; 94 months vs. 55.4 months, P=0.007) and overall survival (OS; 100 months vs. 70.5 months, P=0.031) compared with other antidiabetogenic agents 169. Despite the impressive results of this retrospective trial, only four ongoing clinical trials (ClinicalTrials.gov) are evaluating the effects of metformin with standard systemic chemotherapy in the settings of relapsed and refractory NHL (Table 6).

The combined increase in the occurrence of diabetes, obesity, and cancer has created a significant problem in which complex disease pathophysiologies are intertwined. Dissection of the epidemiological and molecular links between these diseases has revealed novel target molecules and new treatment opportunities. In conjunction with interdisciplinary research, epidemiological and preclinical data support that metformin benefits select patients with solid tumors and hematological tumors; however, strict clinical trials are required to pinpoint patients who might benefit from metformin combinations. Thus, we need to assess whether the anticancer effects of metformin depend on metabolic variables, such as diabetes, BMI, insulin resistance, and obesity-related inflammation.

## AUTHOR CONTRIBUTIONS

Cunha Júnior AD and Pericole FV wrote the initial drafts of the manuscript, and Carvalheira JB revised the manuscript.

## Figures and Tables

**Table 1:** Relative risks associated with overweight and obesity and percentage of cases attributable to overweight and obesity in the United States, the European Union, and Brazil.

Type of cancer	RR* BMI 25-30 kg/m2	RR* BMI>30 kg/m2	FAP* (%) for the US (30, 31)	FAP (%) for the EU (22, 32-34)	FAP (%) for BR (35)
Colon cancer (men)	1.5	2.0	35.4	27.5	43.8
Colon cancer (women)	1.2	1.5	20.8	14.2	25.3
Breast cancer (postmenopausal women)	1.3	1.5	22.6	16.7	17.3
Endometrial cancer	2.0	3.5	56.8	45.2	44.8
Kidney (kidney cells) cancer	1.5	2.5	42.5	31.1	31.3(W)/35.6(M)
Adenocarcinoma of the esophagus	2.0	3.0	53.4	42.7	60.2(W)/72.8(M)
Pancreatic cancer	1.3	1.7	26.9	19.3	24.7(W)/33.9(M)
Hepatocellular carcinoma	ND*	1.5-4	ND*	ND*	23.9(W)/25.9(M)
Gall bladder	1.5	2.0	35.5	27.1	18.2(W)/6.6(M)
Cardiac adenocarcinoma	1.5	2.0	35.5	27.1	62.2(W)/65.5(M)

*RR: relative risk, FAP: fraction attributable to population, BMI: body mass index, ND: not determined, W: women, M: men. USA: United States of America, EU: Europe; BR: Brazil.

**Table 2:** Incidence of hematological malignancies in obese patients.

Hematologic malignancy	Patient cohort and study
Lymphoma	Meta-analysis: RRs for a 5 kg/m(2) increase in BMI were 1.07 (95% confidence interval [CI], 1.04-1.10) for NHL incidence (16 studies, n=17,291 cases) and 1.14 (95% CI, 1.04-1.26) for NHL mortality (five studies, n=3407 cases) (7).
MM	Meta-analysis: RRs were 1.12 (95% CI, 1.07-1.18) for overweight individuals and 1.21 (95% CI, 1.08-1.35) for obese individuals; a total of 15 cohort studies on MM incidence and five studies on MM mortality were included (8).
Leukemia	Meta-analysis: RRs of leukemia were 1.14 [95% CI, 1.03-1.25] for overweight individuals (BMI 25-30 kg/m(2)) and 1.39 (95% CI, 1.25-1.54) for obese individuals (BMI >or= 30 kg/m(2)); a total of 9 cohort studies with data on BMI or obesity in relation to the incidence of leukemia were included (6).
Subtypes of leukemia	Meta-analysis: RRs associated with obesity were 1.25 (95% CI, 1.11-1.41) for CLL, 1.65 (95% CI, 1.16-2.35) for ALL, 1.52 (95% CI, 1.19-1.95) for AML and 1.26 (95% CI, 1.09-1.46) for CML; a total of 4 studies reporting results on subtypes of leukemia were included (6).

ALL: acute lymphocytic leukemia, AML: acute myeloid leukemia, BMI: body mass index, CLL: chronic lymphocytic leukemia, CML: chronic myeloid leukemia, MM: multiple myeloma NHL: Non-Hodgkin Lymphoma, RR: relative risk.

**Table 3:** Meta-analysis on the relative risk (RR) of cancer in different organs in patients with diabetes.

Type of cancer (author and year)	Number of evaluated studies	RR (95% CI*)
Colon (Luo et al. 2012) (36)	24	1.27 (1.14-1.42)
Breast (Boyle et al. 2012) (37)	40	1.27 (1.16-1.39)
Pancreatic (Ben et al. 2011) (38)	35	1.94 (1.66-2.27)
Prostate (Long et al. 2012) (42)	7	2.82 (1.73-4.58)
Prostate (Hwang et al. 2015) (56)	8	1.20 (1.00-1.44)
Liver (Chen et al. 2015) (43)	21	1.86 (1.49-2.31)
Lung (Zhu et al. 2016) (57)	20	1.28 (1.10-1.49)
Bladder (Fang et al. 2013) (44)	24	1.30 (1.18-1.43)
Ovarian (Lee et al. 2013) (58)	18	1.16 (1.01-1.33)

*CI: confidence interval, RR: relative risk.

**Table 4:** Meta-analyses and observational and case-control studies on the risk of cancer in organs of patients with diabetes treated with metformin.

Type of cancer (author and year)	Type of study and patient cohort	RR, HR or OR (95% CI)*
Several (Evans et al. 2005) (2)	Case-Control/2829	OR 0.86 (0.73-1.02)
Several (Zhang et al. 2014) (11)	Meta-analysis of 28 studies/NR	RR combined 0.70 (0.55-0.88)
Several (Gandini et al. 2014) (78)	Meta-analysis of 47 studies/65.540	RR incidence 0.69 (0.52-0.90) RR mortality 0.66 (0.54-0.81)
Several (Yin et al. 2013) (79)	Meta-analysis of 20 studies/13.008	HR OS* 0.66 (0.55-0.79) HR OS* 0.62 (0.46-0.84)
Several (Noto et al. 2012) (76)	Meta-analysis of 24 studies/21.195	RR mortality 0.66 (0.49-0.88) RR incidence 0.67 (0.53-0.85)
Prostrate (Deng et al. 2015) (80)	Meta-analysis of 13 studies/334.532	RR incidence 0.88 (0.78-0.99) RR mortality 1.07 (0.86-1.32)
Prostrate (Raval et al. 2015) (81)	Meta-analysis of 9 studies/8284	HR cancer-specific mortality 1.22 (0.58-2.56)
Lung (Sakoda et al. 2015) (82)	Retrospective observational/47.351	HR adenocarcinoma 0.69 (0.40-1.17) HR small cell carcinoma 1.82 (0.85-3.91)
Lung (Lin et al. 2015) (83)	Retrospective observational/750	HR in favor of metformin use EC IV 0.80 (0.71-0.89)
Lung (Zhu et al. 2015) (45)	Meta-analysis of 8 studies/17.997	RR 0.84 (0.73-0.97)
HNC* (Sandulache et al. 2014) (84)	Retrospective observational/162	OR OS DM vs. non-DM 2.23 (0.9-5.6; P*=*0.04) Lower locoregional recurrence (P*=*0.04)
HNC* (Yen et al., 2014) (85)	Retrospective observational/290	The incidence of CCP was 0.64x lower in the metformin group (pcts>65a had lower risk reduction of CCP)
Breast (Col et al. 2012) (86)	Meta-analysis of 7 studies/17.997	OR 0.83 (0.71-0.97)
Breast (Jiralerspong et al. 2012) (87)	Retrospective/2.529	OR pCR* 2.95 (P=0.04) pCR 24% (met*) x 8% (non-met*)
Breast (Xu et al. 2015) (88)	Meta-analysis of 11 studies/5.464	HR OS* 0.53 (0.39-0.71)
Pancreatic (Wang et al. 2014) (89)	Meta-analysis of 11 studies/764.195	RR 0.63 (0.46-0.86)
Colon (Lee et al. 2012) (90)	Case-Control EC IV/106	HR SLD 0.024 (0.001-0.435) HR OS 0.809 (0.094-6.959)
Colon (Fransgaard et al. 2016) (91)	Retrospective observational/30,493	HR DM vs. non-DM 1.12 (1.06-1.18) HR met vs. insulin 0.85 (0.73-0.93)
Colon (Mei et al. 2014) (92)	Meta-analysis of 6 studies/2.461	HR OS 0.56 (0.41- 0.77) HR OS-specific 0.66 (0.50-0.87)
Endometrial (Nevadunsky et al. 2014) (93)	Retrospective observational/985	HR OS met vs. non-met 0.54 (0.30-0.97)
Ovarian (Dilokthornsakul et al. 2013) (94)	Systematic review of 4 studies/	OR 0.57 (0.16-1.99).
Ovarian (Romero et al. 2012) (95)	Retrospective observational/341	HR disease recurrence met vs. non-met 0.38 (0.16. 0.90)
Ovarian (Kumar et al. 2013) (96)	Retrospective case-control/72 cases and 143 controls	HR OS non-met vs. met 2.2 (1.2-3.8)
Liver (Zhang et al. 2012) (97)	Meta-analysis of 5 studies/105.495	OR prevention 0.38 (0.24-0.59)
Liver (Ma et al. 2012) (98)	Meta-analysis of 11 studies/3452	OR prevention 0.38 (0.24-0.59)

*DM: diabetes mellitus, HNC: head and neck cancer, met: metformin, pCR: pathologic complete response, HR: hazard ratio, OR: odds ratio, OS: Overall survival, RR: relative risk.

**Table 5:** Ongoing clinical studies with metformin in MM.

Type of disease	Disease status	Type of study	Associated drugs	Country of study	ClinicalTrials.gov identifier
MM* or CLL*	Recurrent/refractory	Pilot	Ritonavir	USA*	NCT02948283
MM*	Recurrent/refractory	Phase II	High doses of dexamethasone	BR*	NCT02967276

*MM: multiple myeloma, CLL: chronic lymphocytic leukemia, USA: United States of America, BR: Brazil.

**Table 6:** Ongoing clinical studies with metformin in leukemias and lymphomas.

Type of disease	Disease status	Type of study	Associated drugs	Country of study	ClinicalTrials.gov identifier
CLL*	Relapsed and untreated disease	Pilot	None	USA*	NCT01750567
CLL* or MM*	Recurrent/refractory	Pilot	Ritonavir	USA*	NCT02948283
ALL	Relapsed	Phase I	Vincristine, dexamethasone, doxorubicin, and PEG-asparaginase	USA*	NCT01324180
AML*	Relapsed and refractory	Phase I	Cytarabine	USA*	NCT01849276
NHL* other solid tumors	Relapsed and refractory to other treatments	Phase I	Sirolimus	USA*	NCT02145559
NHL* other solid tumors	Relapsed and refractory to other treatments	Phase I	Temsirolimus	Canada	NCT00659568
DLBCL*	Previously, untreated	Phase II	R-CHOP	USA*	NCT02531308
DLBCL*	Double-hit lymphoma	Phase II	DA-EPOCH-R	USA*	NCT02815397

*ALL: acute lymphocytic leukemia, AML: acute myeloid leukemia, CLL: chronic lymphocytic leukemia, DA-EPOCHR: dose-adjusted etoposide + prednisone + vincristine + cyclophosphamide + doxorubicin + rituximab, MM: multiple myeloma, NHL: non-Hodgkin lymphoma, DLBCL: diffuse large B cell lymphoma, R-CHOP: rituximab + cyclophosphamide + doxorubicin + vincristine + prednisone, USA: United States of America.
